# B1 siRNA Increases *de novo* DNA Methylation of B1 Elements and Promotes Wound Healing in Diabetic Rats

**DOI:** 10.3389/fcell.2021.802024

**Published:** 2022-01-19

**Authors:** Sakawdaurn Yasom, Wilunplus Khumsri, Papatson Boonsongserm, Nakarin Kitkumthorn, Preecha Ruangvejvorachai, Apasee Sooksamran, Rujira Wanotayan, Apiwat Mutirangura

**Affiliations:** ^1^ Center of Excellence in Molecular Genetics of Cancer and Human Disease, Department of Anatomy, Faculty of Medicine, King Chulalongkorn Memorial Hospital, Chulalongkorn University, Bangkok, Thailand; ^2^ Interdisciplinary Program of Biomedical Sciences, Graduate School, Chulalongkorn University, Bangkok, Thailand; ^3^ Department of Oral Biology, Faculty of Dentistry, Mahidol University, Bangkok, Thailand; ^4^ Department of Pathology, Faculty of Medicine, Chulalongkorn University, Bangkok, Thailand; ^5^ Department of Radiological Technology, Faculty of Medical Technology, Mahidol University, Nakhon Pathom, Thailand

**Keywords:** B1 siRNA, DNA methylation, DNA hypomethylation, B1 element, DNA damage, RNA-directed DNA methylation, wound healing, diabetic rat

## Abstract

Alu (B1 in rodents) hypomethylation, commonly found in diabetes mellitus patients, increases DNA damage and, consequently, delays the healing process. Alu siRNA increases Alu methylation, reduces DNA damage, and promotes cell proliferation.

**Aim:** To explore whether B1 siRNA treatment restores B1 hypomethylation, resulting in a reduction in DNA damage and acceleration of the healing process in diabetic rat wounds.

**Methods:** We generated splinted-excisional wounds in a streptozotocin (STZ)-induced type I diabetic rat model and treated the wounds with B1 siRNA/Ca-P nanoparticles to generate *de novo* DNA methylation in B1 intersperse elements. After treatment, we investigated B1 methylation levels, wound closure rate, wound histopathological structure, and DNA damage markers in diabetic wounds compared to nondiabetic wounds.

**Results:** We reported that STZ-induced diabetic rat wounds exhibited B1 hypomethylation, wound repair defects, anatomical feature defects, and greater DNA damage compared to normal rats. We also determined that B1 siRNA treatment by Ca-P nanoparticle delivery restored a decrease in B1 methylation levels, remedied delayed wound healing, and improved the histological appearance of the wounds by reducing DNA damage.

**Conclusion:** B1 hypomethylation is inducible in an STZ-induced type I diabetes rat model. Restoration of B1 hypomethylation using B1 siRNA leads to increased genome stability and improved wound repair in diabetes. Thus, B1 siRNA intervention may be a promising strategy for reprogramming DNA methylation to treat or prevent DNA damage-related diseases.

## Introduction

Diabetes is a life-threatening disease that causes systemic tissue damage and multiorgan dysfunctions, mainly chronic hyperglycemia. Kerner and Brückel (2014) Diabetic ulceration is one of the most common chronic wounds that has more than 70% recurrence in diabetic patients in 5 years of treatment (Riedel et al., 2020; Reiber et al., 1998). Impaired wound healing has become a predominant complication in diabetic patients who eventually have a high risk of limb amputation (Nunan et al., 2014). The imbalance of oxidative production and suppression due to prolonged hyperglycemia directly affects cellular signaling, cellular functions, and cell components, such as DNA strands (Newsholme et al., 2016). Previous studies have reported the association between diabetic conditions and DNA lesions (8-oxo-deoxyguanosine, 8-OHdG; and *γ*-H2A.X) in clinical trials and animal studies (Lee and Chan, 2015; Algire et al., 2012). Hyperglycemia-derived DNA damage in diabetes influences cell proliferation, migration, and functions in various cell types in wound areas, directly affecting the healing process (Davis et al., 2018; Blakytny & Jude, 2006). Correspondingly, irrecoverable DNA damage in wound-healing phases perpetuates a delayed healing process, leading to defective fibroblast maturation, fibrosis deposition, re-epithelialization, and formation of new vessel and granulation tissue in diabetes (Blakytny and Jude, 2006; Leoni et al., 2015). The underlying mechanism of DNA damage-induced impaired wound healing has been studied, and an alteration of epigenetic modification, such as DNA methylation in Alu elements, has been proposed.

Alu elements in humans (B1 elements in rodents) are the most abundant retrotransposon or short intersperse elements (SINEs), containing approximately 40–45% of the human and rodent genomes (Kramerov and Vassetzky, 2005). Alu or B1 elements are located in noncoding regions and are primarily methylated, termed Alu (B1) methylation, to form heterochromatin for maintaining genome stability (Kramerov and Vassetzky, 2005). Recent studies have focused on the DNA methylation pattern, a predominantly epigenetic modification that confers a methyl group to cytosine-phosphate-guanine (CpG) sites along the genome, in age-related conditions (López-Otín et al., 2013; Brunet and Berger, 2014). Changes in the DNA methylation pattern caused by a decrease in methylated CpG islands, which is known as DNA hypomethylation, in genome-wide and intergenic regions distinctively occur during the aging process (Jintaridth and Mutirangura, 2010; Jintaridth et al., 2013). Under certain aging-induced conditions, such as diabetes, Alu hypomethylation is correlated with prediabetic conditions, type II diabetes, and osteoporosis, and it is correlated with the presence of a high rate of DNA damage (Thongsroy et al., 2017). In addition, our laboratory group has reported that the Alu hypomethylation levels in type 2 diabetic patients are strongly correlated with high fasting blood sugar and HbA1C compared to age-matched normal individuals, suggesting that genome-wide hypomethylation might be an implicit contributor to genome instability in diabetes (Thongsroy et al., 2017). Notably, a relevant study has provided evidence that modulation of Alu methylation through *de novo* methylation-mediated Alu small interfering ribonucleic acids (siRNAs) using a mechanism of RNA-directed DNA methylation (RdDM) occurs in human cells (Chalertpet et al., 2019). Moreover, *de novo* Alu methylation by Alu siRNA transfection prevents 8-OHdG and AP site DNA damage, enhances cell growth, and increases cellular tolerance to DNA damage agents in human cell lines (Patchsung et al., 2018). However, focusing on DNA hypomethylation treatment in Alu or B1 repetitive elements in diabetes is inevitably limited (den Dekker et al., 2019; Pastar et al., 2021). Based on the collective evidence showing the relationship between Alu (B1) hypomethylation and DNA damage accumulation in diabetes, we aimed to elucidate the possibility of diabetic wound treatment by reprogramming B1 hypomethylation and strengthening genome integrity to enhance cell proliferation in a preclinical study.

We utilized a streptozotocin (STZ)-induced type I diabetic rat model, which is widely used and well accepted to mimic type I diabetes and successfully demonstrates characteristics of diabetes with increased DNA damage (Furman, 2015; King, 2012). We transfected B1 siRNA or plasmid DNA into target cells using calcium-phosphate (Ca-P) nanoparticles. These nanoparticles act as a nonviral vector to allow inorganic gene transfer, and their composition pre-exists in cells and tissues with biocompatibility and biodegradation features (Zhao et al., 2014; Yin et al., 2014). Due to the low immunogenicity and toxicity of inorganic Ca-P coatings, these nanoparticles are suitable gene delivery systems and provide sufficient transfection efficiency for *in vivo* studies (Ramamoorthi and Narvekar, 2015).

Herein, we hypothesized that topical B1 siRNA treatment promotes wound healing in diabetic rats by attenuating DNA damage and ameliorating the anatomical appearance of the wound. Thus, we investigated whether B1 siRNA delivered by Ca-P nanoparticles efficiently restores B1 hypomethylation in diabetic rat wounds. In conclusion, we found that the increased B1 methylation levels in diabetic wound DNA significantly promotes the healing process by exhibiting improved histopathologic scores and a significant decrease in DNA damage as indicated by 8-OHdG and *γ*-H2A.X.

## Materials and Methods

### B1 siRNA and Plasmid Control Preparation

The B1 siRNA [5′-AGU​UUC​UCU​GUG​UAA​CAG​CCC-3′ (sense) and 5′-GCU​GUU​ACA​CAG​AGA​AAC​UCU-3′ (antisense)] sequences were designed using siDirect 2.0 siRNA design software (Naito and Ui-Tei, 2012) and synthesized by U2Bio Co., Ltd. (Korea). The pLenti-C-mGFP-P2A-Puro (scramble-GFP) plasmid (Origene Technologies, Inc., CA, United States) containing green fluorescent protein (GFP) was utilized as an *in vitro* transfection control, and the pcDNA 3.1 (+) plasmid containing a FLAG sequence (Invitrogen, United States) was used as a transfection control plasmid in a rat wound model. Each plasmid was transformed into competent *E. coli* (DH5*α*) (Invitrogen, United States). After selective bacterial culture, the plasmids were extracted and purified using the GeneJet Plasmid Maxiprep Kit (Thermo Scientific, MA, United States) according to the manufacturer’s instructions. The purity (A260/A280) and concentration of the plasmids were determined using a Thermo Scientific™ NanoDrop 2000 spectrophotometer.

### B1 siRNA and Plasmid Delivery Using Ca-P Nanoparticles *in vitro* and *in vivo*

To deliver the B1 siRNA or the plasmid control into the target cells, each type of genetic material was coated with the nanoparticle solution as previously described by Zhao et al. (2014) with some modifications before topical administration (Zhao et al., 2014). The 100 nM of working B1 siRNA was prepared from 100 µM stock B1 siRNA, synthesized by U2Bio Co., Ltd. (Korea), and incorporated with Ca-P nanoparticle before a topical application (100 µl of B1siRNA/Ca-P nanoparticle solution/wound/day). The most effective plasmid ratio to nanoparticle solution for transfection was 5 µg plasmid in 100 µl of nanoparticle solution. The nanoparticle solution was comprised of 50 µl of a mixture of 0.5 M calcium chloride (CaCl_2_) solution (Merck Millipore, United States) and 5 µg of plasmid DNA as well as 50 µl of a mixture of 0.01 M sodium carbonate (Na_2_CO_3_) solution (Merck Millipore, United States) and 0.01 M sodium dihydrogen phosphate monohydrate (NaH_2_PO_4_·H_2_O) solution (Merck Millipore, United States). A molar ratio of CO_3_^2-^/PO_4_^3-^ of 31:1 was used. First, the plasmid was mixed with 16 µl of 0.5 M calcium chloride (CaCl_2_) solution, and the final volume was adjusted to 50 µl using sterile dH_2_O. Then, the plasmid DNA-calcium complex was added to 50 µl of a mixture of sodium carbonate solution (16 µl), sodium dihydrogen phosphate monohydrate solution (16 µl), and sterile dH_2_O (34 µl). The nanoparticle-coated plasmid solution was prepared before use.

## Wound Healing in an Animal Study

### Diabetic Rat Model

Male Wistar rats (6 weeks old and 150–180 g) were obtained from the National Laboratory Animal Center, Mahidol University, Bangkok, Thailand. The Institutional Animal Care and Use Committee (IACUC) approved the animal use protocol at the Faculty of Medicine, Chulalongkorn University (approval number: 006/2,561 in September 2018). Rats were maintained on a control 12:12 h light:dark cycle and *ad libitum* fed standard normal chow and water. Animals were allowed to acclimatize for 7 days before type 1 diabetes induction. Rats were then randomly divided into two groups and intraperitoneally injected with 1) a single dose of 65 mg/kg body weight STZ (Sigma–Aldrich, United States) dissolved in 50 mM sodium citrate buffer (Alfa Aesar, United States) and 2) 50 mM sodium citrate buffer (2 mL/kg body weight) (Furman, 2015; King, 2012). After 7 days of STZ induction, the fasting blood sugar (FBS) levels were monitored in both groups using a glucometer and blood glucose strips (ACCU-CHEK^®^ Roche, Germany). The STZ-induced rats with FBS greater than 250 mg/dL were considered diabetic rats, whereas the rats with FBS lower than 150 mg/dL were considered nondiabetic rats (Figure 3).

### Excisional Wound Protocol

Two paired full-thickness excisional wounds were created at the dorsa of rats using an 8 mm biopsy punch and splinted with silicone rings (Davidson et al., 2013; Yao et al., 2014). Diabetic and nondiabetic rats were further subdivided into two groups and treated with nanocoated B1 siRNA and normal saline solution (NSS), and the NSS-treated group represented the standard wound dressing in this study. The nondiabetic and diabetic wounds were dressed daily and treated with each type of intervention for 14 days. The wound area was measured at days 0, 3, 5, 7, 10, and 14 after the treatment and was reported as the percent wound closure rate using the following formula: percent wound closure rate = [(wound area *day 0*—wound area *day n*)/wound area *day 0*] x 100 (day *n* representing days 3, 5, 7, 10, or 14). To investigate the expression of the genetic materials using Ca-P nanoparticles in rat wounds, the nanocoated pcDNA 3.1 (+) plasmid control with the FLAG-tagged protein sequence was topically applied to the wounds. After 14 days of the complete healing process, all rats were sacrificed, and the wound areas were excised and immediately collected in DNAzol reagent (Invitrogen, United States) for measurement of DNA methylation levels and in 10% formalin buffer for histological determination and immunohistochemistry staining.

## DNA Extraction and Bisulfite DNA Modification

Rat wound DNA in DNAzol reagent was extracted using a standard protocol of tissue genomic DNA extraction (Chomczynski et al., 1997). Briefly, 25–30 mg of rat wound tissue in 1 ml of DNAzol reagent was homogenized in a tissue homogenizer (IKA ULTRA-TURRAX dispersers, Sigma–Aldrich, United States) followed by a tissue genomic DNA extraction protocol according to the manufacturer’s instruction. The extracted rat genomic DNA was dissolved in 8 mM NaOH, and genomic DNA concentration and purity were detected by a NanoDrop 2000 spectrophotometer (Thermo Scientific™, United States). The rat genomic DNA was stored at −20°C until bisulfite DNA modification was performed. Rat genomic DNA (400 ng) was subjected to sodium bisulfate DNA modification according to the manufacturer’s instruction manual (EZ DNA Methylation-Gold™ Kit, Zymo Research, CA, United States).

## Measurement of B1 Methylation by B1-Combined Bisulfite Restriction Analysis

To determine the DNA methylation levels of B1 elements in the rat wound area, bisulfite-treated rat wound DNA was subjected to B1-PCR amplification using a PCR mixture, containing 1X PCR buffer (Qiagen, Germany), 1 mM MgCl_2_ (Qiagen, Germany), 0.2 mM dNTPs (Promega, United States), 25 U HotStarTaq DNA polymerase (Qiagen Germany), and 0.2 µM B1-Forward (5′-YGYAYGYYTTTAATYYYAGYAAT-3′) and B1-Reverse (5′-CCCTRRCTRTCCTRRAACTCA-3′) primer pairs. B1-PCR was amplified using the following protocol to generate a 98 bp amplicon: 95°C for 15 min; 35 cycles of 95°C for 45 s, 53°C for 45 s, and 72°C for 45 s; and final extension of 72°C for 10 min. For B1-COBRA, the B1-PCR products were subsequently subjected to the digestion of 2 units of TaqI restriction enzyme (Thermo Scientific, MA, United States) at 65°C for 16 h. The digested B1-PCR products were then analyzed by 8% acrylamide gel electrophoresis and stained with SYBR green (Lonza, Basel, Switzerland). Rat dermal fibroblast (RDF) DNA was subjected to bisulfite DNA modification, B1-COBRA, and TaqI digestion by similar protocols as the experimental samples, and it was used as a rat genomic internal control DNA. The methylation of B1-COBRA was assessed by detection of band intensity using Image Quant software (GE Health care^®^, United Kingdom) (Thongsroy et al., 2017; Patchsung et al., 2018).

## Determination of B1 Methylation Level

The B1 methylation level was assessed by calculating the digested B1-PCR products after TaqI digestion, and four different product sizes (98, 78, 54, and 44 bp) were detected and used to differentiate the B1 methylation status. Two forms of B1 methylation were reported, including B1 loci with methylated CpGs (mC) and B1 loci with unmethylated CpGs (uC). The formula of B1 methylation calculation was designated by the band intensity per amplicon size (bp) as follows: A = 98/98, B = 78/74, C = 54/54, and D = 44/42. The band intensities of each sample were normalized by using the average intensity of the internal RDF DNA as mentioned above. The B1 methylation level was calculated from the percentage of methylated band intensity divided by the total mC and uC band intensities as follows [(A + C + D)/2 (A + B + D) x 100] (Thongsroy et al., 2017; Patchsung et al., 2018).

### Histopathological Analysis

After wound collection, the wounds were fixed in 10% neutral buffered formalin for at least 48 h. The tissues were then dehydrated and paraffin-embedded before 3 µm thick tissue sectioning by a microtome. Subsequently, the tissue sections were stained with H&E and Giemsa for tissue histopathology and immune cell infiltration, respectively. The histopathological evaluation was blindly performed and interpreted by two pathologists. Tissue granulation and re-epithelization were investigated in the observed areas of healing wounds and reported as the overall histological score as follows: 1 = normal tissue; 2 = mature fibroblasts; 3 = immature fibroblasts; 4 = mild inflammation; and 5 = granulation tissue.

## Immunohistochemistry for FLAG, 8-OHdG, and *γ*H2A.X Staining

Paraffin-embedded sections (3 μm) were deparaffinized and subjected to antigen retrieval by proteinase K (DAKO, CA) incubation for 2 min. To elucidate the transfection efficiency of Ca-P nanoparticles after FLAG-tagged plasmid control/Ca-P transfection in diabetic wounds, the wound sections were incubated with 1:500 anti-FLAG (DYKDDDDK) rabbit monoclonal antibody (Cell Signaling, MA) followed by incubation with HRP-conjugated anti-rabbit secondary antibody (DAKO, CA). For DNA damage determination, the wound sections were incubated with a 1:8,000 dilution of polyclonal goat anti-8-OHdG antibody (Merck Millipore) or a 1:100 dilution of rabbit anti-*γ*H2A.X antibody (Abcam) followed by incubation with HRP-conjugated anti-goat or rabbit secondary antibody (DAKO, CA). Subsequently, the sections were counterstained with hematoxylin.

### Confocal Microscopy

To investigate the efficiency of transfection using nanoparticle-coating solution, Ca-P nanoparticle-coated scramble-GFP plasmids were transfected into HEK293 cells (5 × 10^4^ cells/well) in a 24-well plate 24 h after cell seeding. The transfected HEK293 cells were observed, and images were acquired at 20 × 48 h after transfection using a confocal microscope (ZEISS LSM 800, CARL ZEISS, United States).

## Statistical Analysis

The data were expressed as the mean ± SEM. One-way ANOVA was performed to test the difference between the groups followed by post hoc analysis. *p* < 0.05 was considered statistically significant using GraphPad Prism version 9.

## Results

### Plasmid/Ca-P Nanoparticle Transfection in Cell Culture and Topical Application in a Murine Wound

**FIGURE 1:**
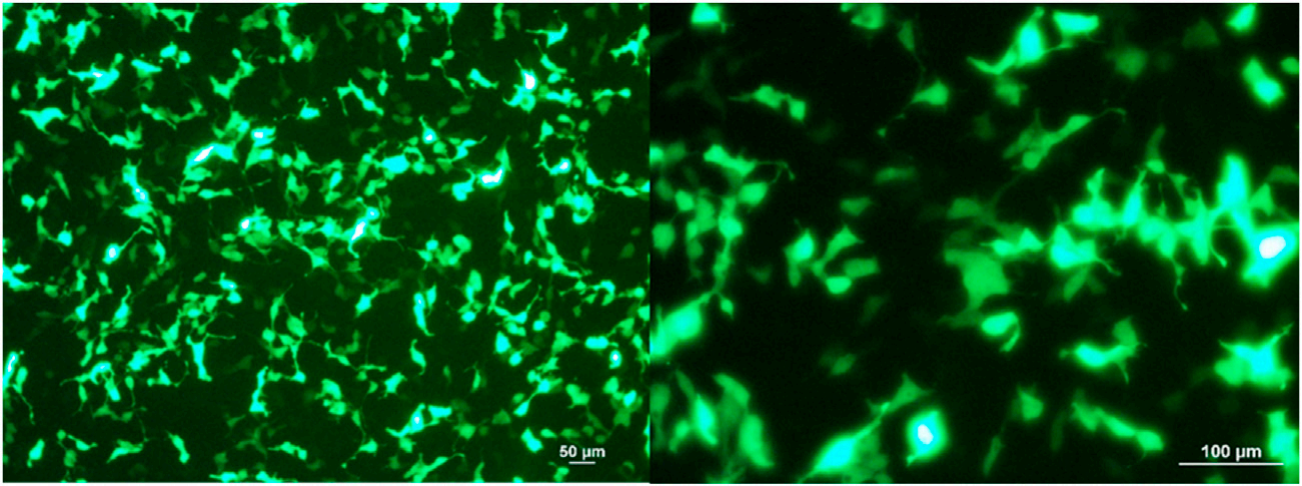
GFP-expressing HEK293 cells after 72 h of plasmid/Ca-P nanoparticle transfection. Cells (5 × 10^4^) were transfected with GFP plasmid-encapsulated Ca-P nanoparticles (5 µg plasmid/100 µl Ca-P nanoparticle solution). At 72 h after transfection, representative images were acquired using a fluorescence microscope at × 10 and × 20 magnifications (Zeiss LSM 800, Carl Zeiss, United States). The green color shows the GFP produced by the transfected HEK293 cells.

**FIGURE 2:**
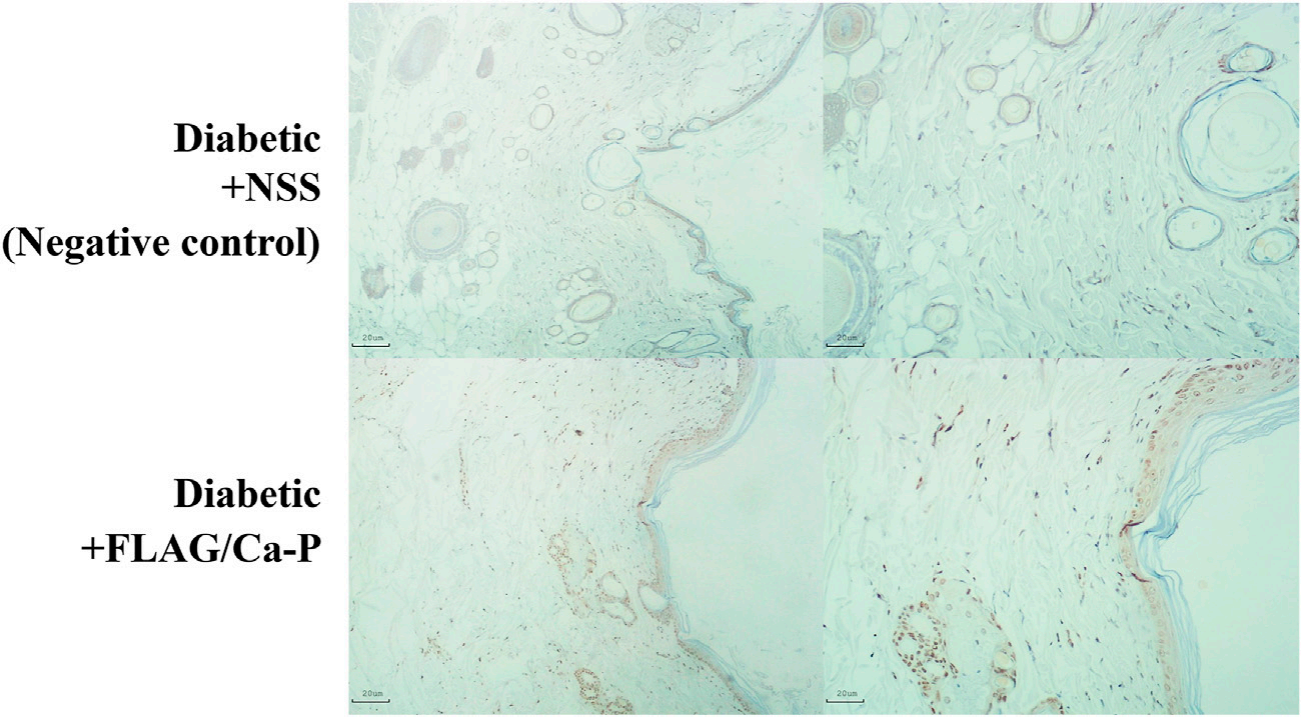
FLAG-expressing cells in diabetic wounds. FLAG-tagged plasmid control (pcDNA3.1)-encapsulated Ca-P nanoparticles (lower panel) were applied daily to excisional wounds in diabetic rats by topical treatment for 14 consecutive days, and the control wounds were treated with NSS (negative control, upper panel). At the end of the study, the wounds were dissected and stained for FLAG protein expression by IHC staining. Positive anti-FLAG staining (brown color, lower panel) was observed in many cell types in the *epidermis*, dermis, and *panniculus carnosus* muscle layer, including mainly mature fibroblasts and keratinocytes, compared to the no transfection control (the NSS-treated wounds). Representative images of diabetic wounds were acquired at ×4 (left panel) and ×10 (right panel) magnifications.

The plasmid-encapsulated Ca-P nanoparticles successfully delivered and expressed green fluorescent protein (GFP) in HEK293 cells and the FLAG protein in a murine excisional wound model. We transfected the GFP plasmid/Ca-P nanoparticle complex into 5 × 10^4^ HEK293 cells, and GFP expression was observed 72 h after transfection (Figure 1). To elucidate the efficiency of Ca-P nanoparticle transfection in murine wounds, FLAG-tagged plasmid (pcDNA3.1)-encapsulated Ca-P nanoparticle complexes were topically applied into excision wounds daily for 14 days. At 14 days after topical treatment, rat wounds were collected to detect FLAG protein expression. Positive anti-FLAG staining was found in various cell types in the wound area (mainly mature fibroblasts), the dermis, *panniculus carnosus* muscle layer, and keratinocytes (Figure 2). Therefore, the Ca-P nanoparticles effectively delivered genetic materials in cell culture and rat wounds by topical administration.

### B1 siRNA Topical Treatment Restores the B1 Methylation Level in Diabetic Wounds

**FIGURE 3:**
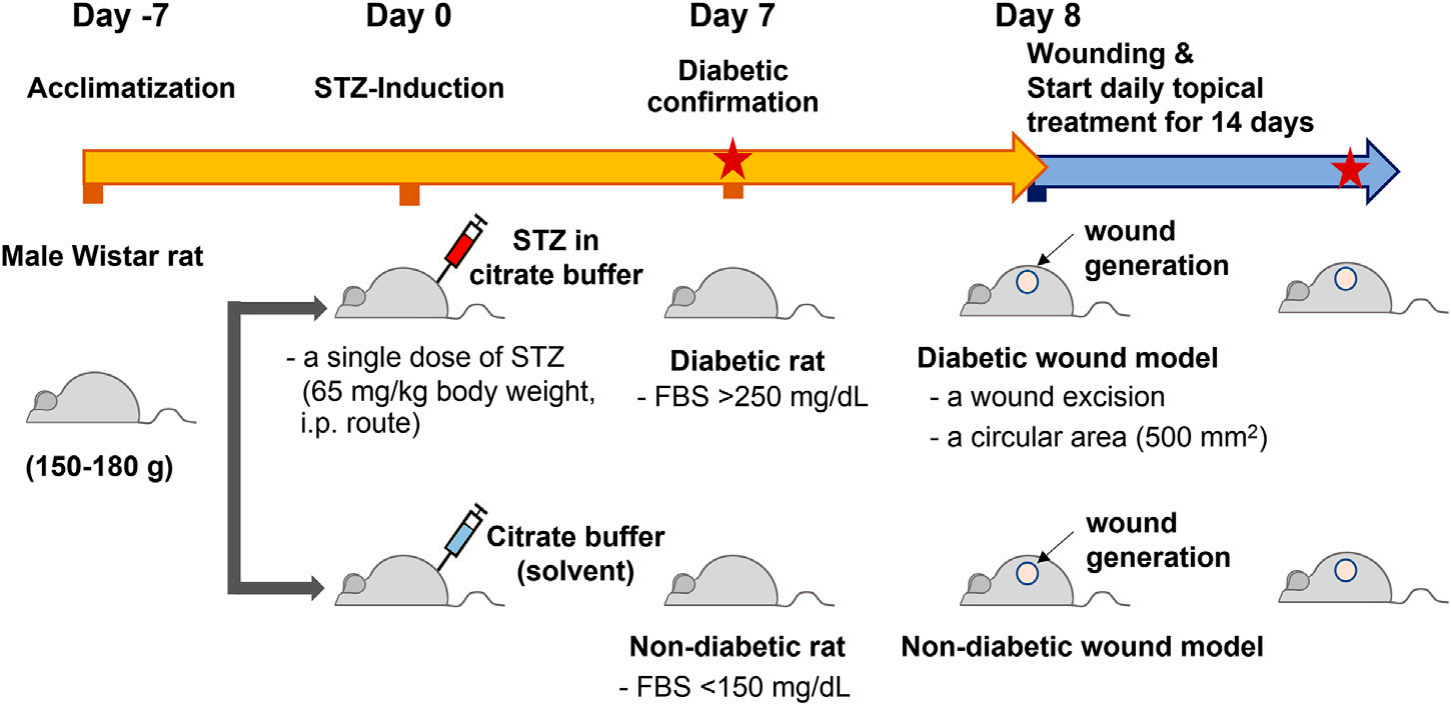
The schematic of experimental design. Rats were allowed to acclimatize for 7 days and measured the baseline FBS levels before they were randomly divided into two groups. The first group was intraperitoneally injected with a single dose of streptozotocin (STZ), 65 mg/kg body weight. The other group was injected with sodium citrate buffer (2 mL/kg body weight, STZ solvent) with the same route. After 7 day STZ induction, the fasting blood sugar (FBS) levels were monitored in both groups. The STZ-induced rats with the FBS greater than 250 mg/dL were designated as a diabetic group, whereas those with the FBS lower than 150 mg/dL were considered nondiabetic groups. The excisional wounds were generated on the rat dorsa on day 8 (day 0 of the treatment). The topical treatment of B1 siRNA or NSS was performed daily for 14 consecutive days until the end of the study, and the FBS levels were detected on day 7 and day 14 of the treatment (Red stars represent the measurement of FBS).

**FIGURE 4:**
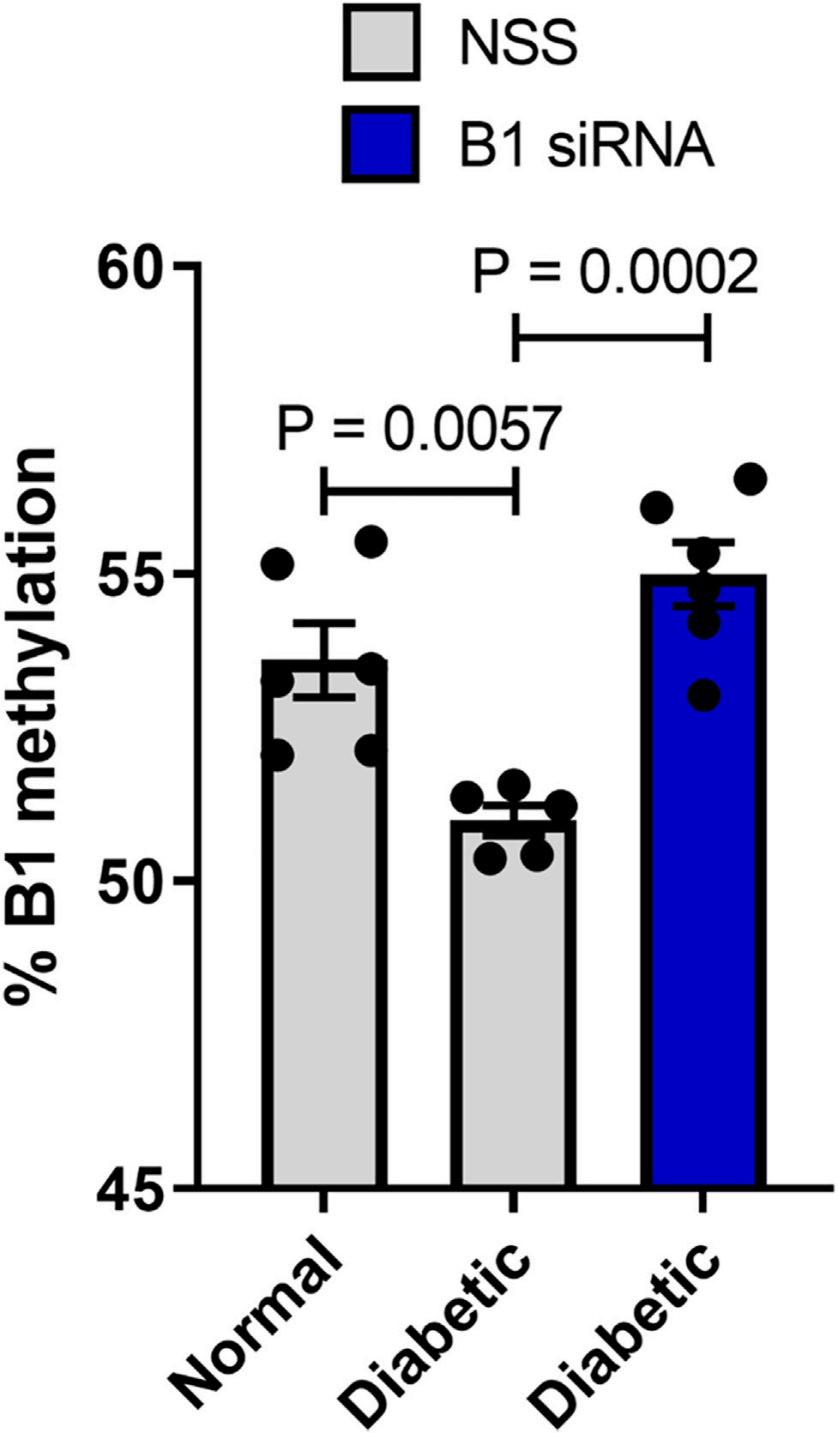
B1 siRNA treatment increases B1 methylation levels in diabetic rat wound DNA. Twenty-one days after STZ induction, rat wound DNA was extracted and modified by the bisulfite method followed by the B1-COBRA technique as described in the Materials and Methods. Diabetic wound DNA showed a significantly decreased B1 methylation level compared to nondiabetic wound DNA. After 14 days of B1 siRNA daily treatment, the B1 methylation levels in B1 siRNA-treated DNA were significantly higher than those in NSS-treated DNA. Data represent the means ± S.E.M. **p ≤ 0.01 and ***p ≤ 0.001 according to one-way ANOVA followed by post hoc analysis (*n* = 5-6 per group).

Altered DNA methylation patterns found in certain gene promoters and intergenic regions are correlated with diabetes and age-related diseases (Thongsroy et al., 2017; Bansal and Pinney, 2017; de Mello et al., 2014). Our group previously reported the association between hypomethylation of SINEs and Alu elements in prediabetic and diabetic patients (Thongsroy et al., 2017). The use of STZ administration to generate a TIDM animal model is widely accepted for preclinical wound-healing studies (King, 2012; Furman, 2015). The schematic of experimental design was described in Figure 3. In our study, the STZ-induced rats exhibited significantly higher levels of FBS 7 days after STZ injection (65 mg/kg rat body weight), and prolonged hyperglycemia was detected in diabetic rats compared to nondiabetic littermates at the beginning, during, and the end of the study (Table 1, Supplementary Figure S1, and Supplementary Table S1). We first explored whether a single dose of STZ induction in a TIDM rat model sufficiently induces hypomethylation of B1 elements. We demonstrated that STZ-induced diabetic rat DNA exhibited a significant decrease in B1 methylation levels at day 21 after STZ induction compared to normal (nondiabetic) wound DNA (*p* = 0.0057) (Figure 4). To investigate the capability of B1 siRNA to increase the level of B1 methylation, the B1 methylation status of diabetic rat wound DNA was measured between the B1 siRNA- and NSS-treated groups at 14 days after the intervention. B1 siRNA-treated wound DNA showed a significantly higher percentage of B1 methylation than the NSS-treated wound DNA (*p* = 0.0002) (Figure 4). Additionally, B1 siRNA treatment provided *de novo* DNA methylation into the rat genome in a specific manner (Supplementary Figures 2A–E). Notably, B1 siRNA-treated nondiabetic rat wounds showed slightly enhanced levels of B1 methylation, but there was no significant difference from the NSS-treated control (*p* = 0.080) (Supplementary Figure 2F). These findings indicated that a single high dose of STZ induced T1DM with B1 hypomethylation in diabetic rat wounds and that topical B1 siRNA treatment successfully restored B1 methylation loss in diabetic wound DNA.

**TABLE 1:** The body weight and fasting blood glucose level of nondiabetic (normal) and diabetic rats at the end of the study. After 7 days of STZ induction, fasting blood glucose levels were measured (>250 mg/dL defined as a diabetic rat), and this group was designated as the diabetic group (*n* = 5 each group). Citrate buffer was injected into the nondiabetic group (FBS<150 mg/dL), which was designated as the normal control group. After wounding, the diabetic wounds were treated daily with NSS or the B1 siRNA/Ca-P nanoparticle complex and were compared to the NSS nondiabetic wound.

Rat model	Nondiabetic	Diabetic	
		NSS treated	B1 siRNA treated
Body weight (g)	278.0 ± 4.1	193.6 ± 6.9***	191.6 ± 5.8***
Fasting blood glucose (mg/dL)	122.6 ± 8.6	424.4 ± 18.4***	410.8 ± 11.1***

Data are presented as means ± S.E.M. ***p < 0.001 significant difference compared with the nondiabetic group.

### Increased B1 Methylation Levels Were Associated With Wound Healing Acceleration by Showing Improved Histopathological Scores and the Lowering of DNA Damage in Diabetic Rats

**FIGURE 5:**
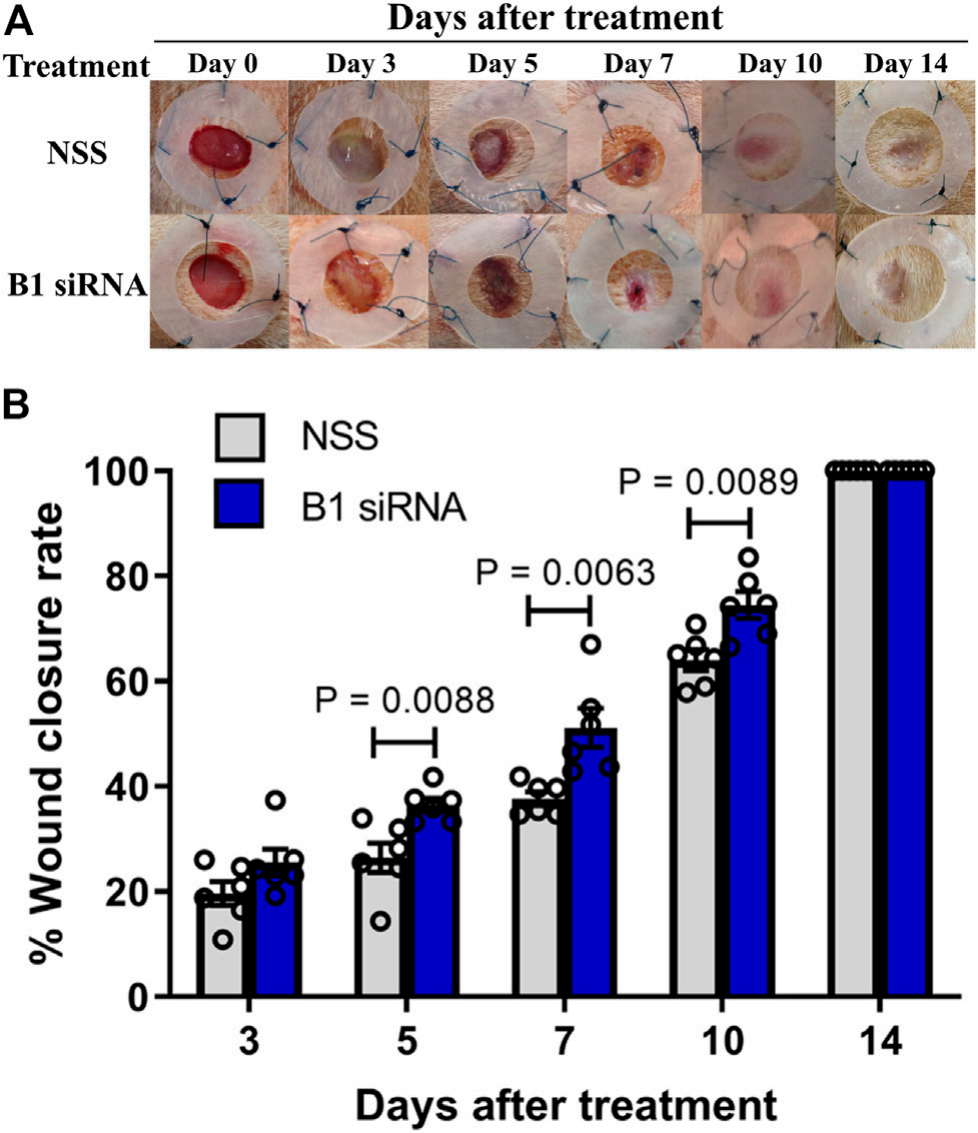
Diabetic wound healing is accelerated by B1 siRNA treatment. **(A)** Images of diabetic rat wounds. Splinted 8 mm excisional wounds at 0, 3, 5, 7, 10, and 14 days after daily treatment with NSS (control, upper panel) or B1 siRNA/Ca-P (lower panel). **(B)** The percentage of wound closure rate after B1 siRNA treatment. The wound areas were measured each day after B1 siRNA (blue bar) or NSS (gray bar) treatment, and the wound closure rate was calculated by comparison with the wound area on day 0 using the formula mentioned in the Materials and Methods. A significantly improved wound healing rate was observed at 5, 7, and 10 days after treatment. Data represent the means ± S.E.M. **p ≤ 0.01 according to one-way ANOVA followed by post hoc analysis (*n* = 5,6 per group).

**FIGURE 6:**
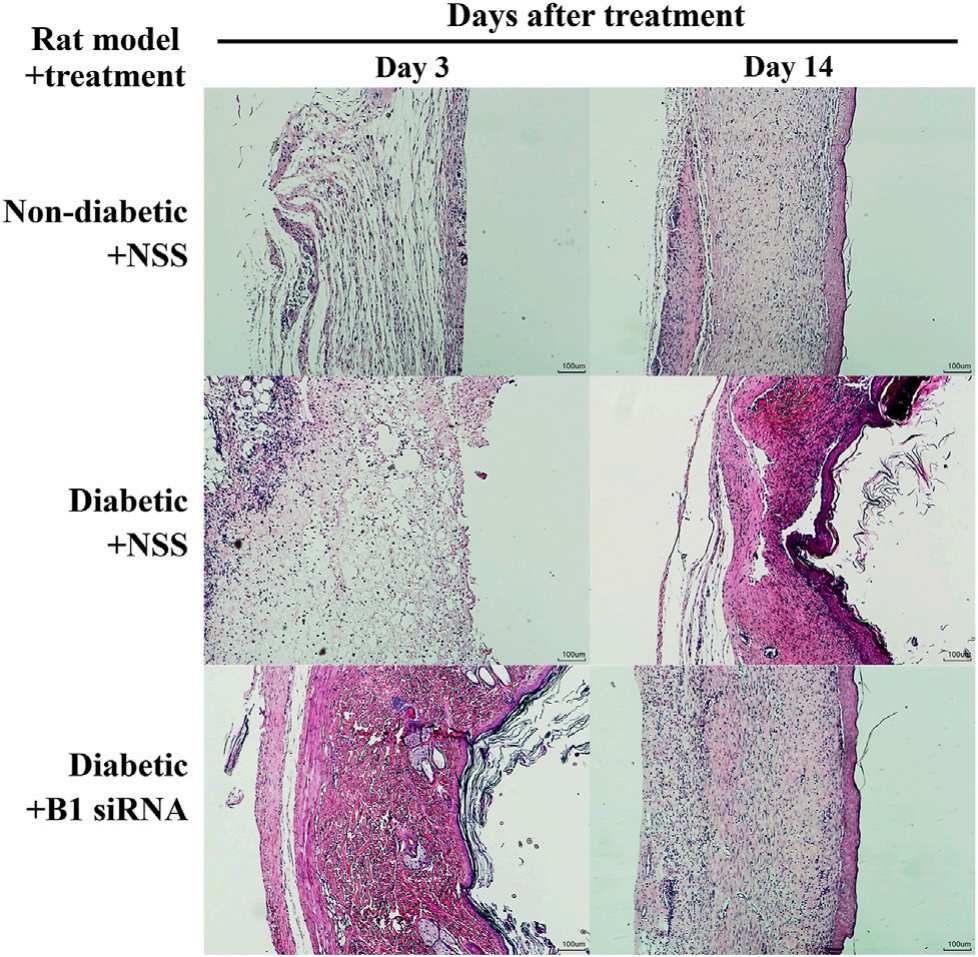
Histopathological parameters are improved and fibroblast proliferation is increased in diabetic wounds after B1 siRNA treatment. H&E staining of wound sections was investigated in a rat model of nondiabetic + NSS (normal control, upper panel), diabetic + NSS (negative control, middle panel), and diabetic + B1 siRNA (treatment, lower panel) at 3 days after treatment and at the end of the study (14 days). B1 siRNA-treated diabetic wounds exhibited enhanced fibroblast proliferation and maturation as well as reduced immune cell infiltration after a treatment period compared to NSS-treated wounds. B1 siRNA treatment demonstrated complete wound closure and similar anatomical appearance of the wounds to nondiabetic (normal) wounds.

Diabetic patients have suffered from delayed wound healing for decades in both developed and developing countries (Öhnstedt et al., 2019; Riedel et al., 2020; Thewjitcharoen et al., 2020; Kosachunhanun et al., 2012). One of the major contributors that causes impaired tissue repair in diabetic wounds is oxidative stress-induced DNA damage, which interrupts the proliferative and inflammatory phases of wound healing (Patel et al., 2019; Rogulj et al., 2017). We hypothesized that the restoration of B1 methylation levels in DNA from diabetic wounds improves the wound-healing process. We investigated the closure of diabetic wounds and reported the percentage of wound closure. Representative images of the wounds in diabetic rats at days 0–14 after wounding are shown in Figure 5A. The wound closure rate demonstrated a higher degree of closure in B1 siRNA-treated wounds than in NSS-treated wounds (Figure 5A). We found that the healing rate of B1 siRNA-treated wounds at days 5, 7, and 10 was significantly higher than that of the NSS-treated wounds (*p* = 0.0088, *p* = 0.0063, and *p* = 0.0089, respectively) (Figure 5B). We confirmed that the higher rate of wound closure in B1 siRNA-treated wounds positively correlated with the anatomical appearance of wound sections by scoring histological parameters (Table 2), and representative images of H&E staining are shown in Figure 6. Treatment with B1 siRNA resulted in the improvement of overall scores that were graded from a composition of mature and immature fibroblasts, an appearance of normal and granulation tissue, and inflammation by demonstrating a significantly lower degree of overall grading than the control-treated diabetic wounds (*p* = 0.0037) (Table 2). In addition, a significant increase in the fibroblast and fibrosis density grades as well as new blood vessel formation was observed in wounds treated with B1 siRNA compared to the control treatment (*p* = 0.0247, *p* = 0.0462, and *p* = 0.0247, respectively). We also investigated the endogenous DNA damage markers, 8-OHdG and *γ*H2A.X, by IHC staining. The IHC staining of 8-OHdG and *γ*H2A.X revealed a significant decrease in the endogenous DNA damage in diabetic wounds treated with B1 siRNA compared to NSS-treated diabetic wounds (*p* = 0.0006 and *p* = 0.0051, respectively) (Table 2). The representative images of 8-OHdG and *γ*H2A.X IHC staining are shown in Supplementary Figures S3, 4, respectively. These findings revealed that treatment with B1 siRNA resulted in the restoration of B1 methylation in diabetic wounds, accelerated wound healing by improving histological parameters (mainly mature fibroblast proliferation), and reduced the DNA damage (as indicated by 8-OHdG and *γ*H2A.X).

**TABLE 2:** Histological parameters of diabetic wounds at day 14 after daily treatment with NSS (*n* = 6) and B1 siRNA (*n* = 6). Histological scores were graded, including overall grading (1 = normal tissue, 2 = many mature fibroblasts, 3 = many immature fibroblasts, 4 = mild inflammation, and 5 = granulation tissue), fibroblasts (0 = absent, 1 = immature, and 2 = mature), fibrosis (0 = absent and 1 = present), new vessel formation (0 = absent, 1 = few, 2 = moderate, and 3 = many), inflammatory infiltration (0 = absent, 1 = few, 2 = moderate, and 3 = many), and DNA damage (8-OHdG and *γ*H2A.X) (0 = absent, 1 = a few, 2 = moderate, and 3 = many).

Histological parameter	NSS-treated	B1 siRNA-treated
Overall grading	2.33 ± 0.21	1.33 ± 0.21**
Fibroblast	0.67 ± 0.21	1.33 ± 0.21*
Fibrosis	0.83 ± 0.17	1.33 ± 0.21*
New vessels	0.33 ± 0.21	1.17 ± 0.31*
Immune cell infiltration		
- Neutrophils	0.17 ± 0.17	0.00 ± 0.00
- Lymphocytes	1.00 ± 0.00	0.83 ± 0.17
- Macrophages	1.17 ± 0.40	0.00 ± 0.17**
- Plasma cells	1.00 ± 0.17	0.83 ± 0.21
DNA damage marker		
- 8-OHdG	2.67 ± 0.21	1.33 ± 0.21***
- γH2A.X	2.50 ± 0.22	1.50 ± 0.22**

AbbreviationNSS-treated; normal saline solution-treated group.

Data are presented as the mean ± SEM. *p < 0.05, **p < 0.01, and ***p < 0.001 indicate significant differences compared to the NSS-treated group.

## Discussion

**FIGURE 7:**
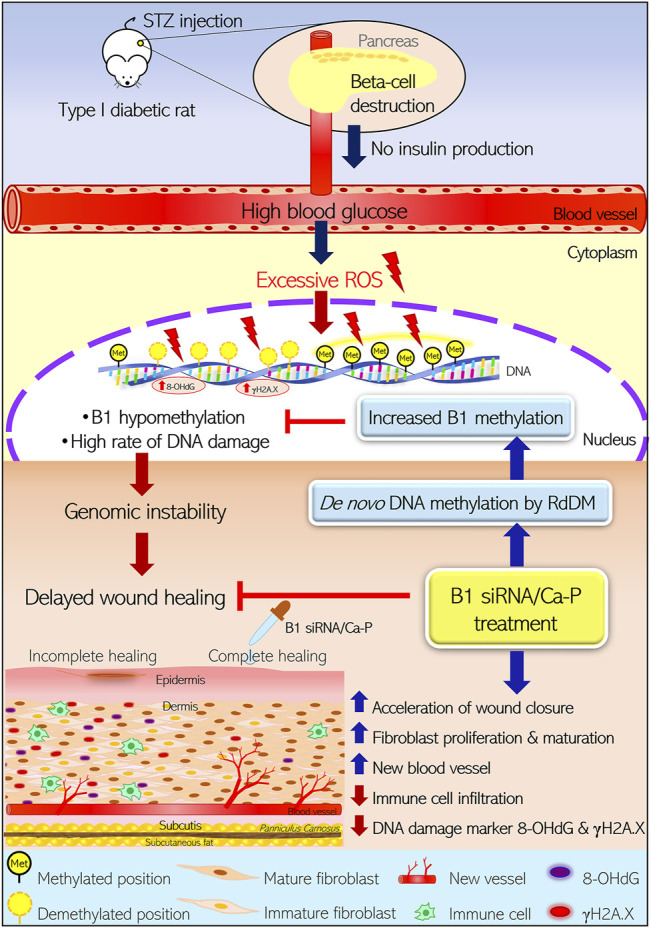
Reprogramming B1 methylation by B1 siRNA transfection restores delayed wound healing in diabetic wounds by limiting DNA damage. STZ-induced type I diabetic rats exhibited high blood glucose due to direct pancreatic *ß*-cell destruction, resulting in no insulin production. Excessive ROS-derived high blood glucose caused oxidative damage of DNA as indicated by 8-OHdG and *γ*H2A.X. STZ-induced diabetic wounds also demonstrated B1 hypomethylation, correlating with a high rate of DNA damage. The accumulation of DNA damage and B1 hypomethylation led to genomic instability and eventually delayed the wound closure rate in diabetic rats. Incomplete healing of wounds resulted in reduced fibroblast maturation and proliferation as well as new blood vessel formation and high immune cell infiltration, which were related to increased DNA damage (8-OHdG and *γ*H2A.X) in the wound bed. In contrast, diabetic wounds treated with B1 siRNA/Ca-P showed an accelerated wound healing rate, correlating with higher fibroblast proliferation and new vessel formation in the wound area compared to diabetic control wounds. In addition, B1 siRNA/Ca-P treatment diminished immune cell infiltration and DNA damage markers, contributing to complete wound healing in diabetic microenvironments. B1 siRNA enhanced *de novo* DNA methylation in B1 elements and restored B1 hypomethylation via the RdDM mechanism. (STZ; streptozotocin, ROS; reactive oxygen species, RdDM; RNA-directed DNA methylation).

Here, we reported that STZ-induced type I diabetic wounds showing B1 hypomethylation and increased DNA damage could be reprogrammed by transfecting B1 siRNA/Ca-P, which improved genome instability and impaired healing (Figure 7). First, our study demonstrated that the type I diabetic rat model induced by a single high dose of STZ injection showed B1 hypomethylation and a high rate of DNA damage, typically characterized as genome instability features. Second, we demonstrated that B1 siRNA caused specific methylation at CpGs in repetitive B1 elements, resulting in a restoration of B1 methylation status, subsequently reducing DNA lesions and strengthening the genome through topical administration in a diabetic rat wound. Finally, the increased B1 methylation contributed to the acceleration of wound healing by decreasing immune cell infiltration and improving histopathologic scores. These findings suggested that abnormalities in epigenetic modifications, particularly DNA hypomethylation, in diabetes can be modulated, providing a promising epigenetic reprogramming technique to be used in preclinical studies for diabetic wound treatment.

Previous studies in mammalian cells and plants have elucidated the mechanism of *de novo* DNA methylation through the action of B1 siRNA, which is called RNA-directed DNA methylation (RdDM) (Chalertpet et al., 2019; Zhang and Zhu, 2011). B1 siRNA transfection increases *de novo* DNA methylation levels that are targeted at interspersed or transposable B1 elements in the rat genome in a specific manner, which is mediated by B1 siRNAs, argonaute 4 (AGO4), and various accessory proteins (Chalertpet et al., 2019). Notably, we observed that B1 siRNA transfection slightly increased the B1 methylation levels after B1 siRNA treatment in normal wounds, but there was no significant difference (*p* = 0.080) between the B1 siRNA treatment and control treatment. B1 elements in normal conditions (not under pathologic conditions) did not present B1 hypomethylation as found in the diabetic rat model. Therefore, a significant difference in B1 methylation levels after B1 siRNA treatment was observed only in diabetic rats when compared to untreated control wounds.

Even though the molecular mechanism whereby B1 (Alu) methylation decreases DNA damage and provides genome strength remains unclear, we proposed several possible mechanisms. First, compelling studies have reported the correlation between methylation in interspersed elements and heterochromatin formation, an epigenetic remark for maintaining genome stability (Baylin et al., 2001). Heterochromatin, the condensed fraction of the genome, may protect DNA against exposure to DNA-damaging agents (Grewal and Jia, 2007), and heterochromatin defects found in an age-associated phenomenon occur prior to accumulative DNA damage (Pegoraro et al., 2009). Therefore, the present study indicated that increased IRS methylation that directly strengthens the genome may result in DNA lesion reduction and subsequently promote cell proliferation in diabetic wounds. Second, we previously reported that the hypermethylated genome retains replication-independent endogenous DNA double-strand breaks (RIND-EDSBs) (Pornthanakasem et al., 2008), which are redefined as youth-associated genomic-stabilizing DNA gaps (Youth-DNA-GAPs) depending on their role (Mutirangura, 2018). The present study suggested that Youth-DNA GAPs may act as epigenetic regulators in reducing DNA lesion accumulation to release DNA tension in the same manner as a gap between two railway lines (Kongruttanachok et al., 2010; Thongsroy et al., 2013; Pongpanich et al., 2014). Finally, increased IRS methylation may improve wound healing by indirectly reducing oxidative stress or the inflammatory response in diabetic wound beds through other mechanisms, consequently reducing DNA damage. While B1 (Alu) siRNA methylated approximately 5% of B1 (Alu) loci, genomic stabilization spanned the whole genome. Therefore, the RIND-EDSB hypothesis is the most likely possible mechanism. Thus, our results showed that B1 hypermethylation in the rat genome accelerated the closure rate of diabetic wounds to a greater extent than that in the untreated group (Figure 5). Correspondingly, our finding agreed with a previous study that transfected Alu siRNA into human cells, leading to *de novo* Alu methylation, resulting in increased DNA damage resistance and enhanced cell proliferation (Patchsung et al., 2018). Similarly, the positive correlation between Alu hypermethylation and catch-up growth in young individuals has been previously reported (Rerkasem et al., 2015). These findings suggest that B1 (or Alu) methylation may be reprogrammed, affecting genome integrity by reducing the accumulation of DNA lesions under some stress conditions.

High blood glucose causes excessive oxidative stress and leads to accumulated DNA damage in diabetic wounds (Newsholme et al., 2016). A major contributor to delayed wound repair in diabetes is a high rate of accumulated DNA damage, which induces cell proliferation defects and coincides with prolonged inflammation associated with carcinogenesis in multiple types of cancer, resulting from a higher mutation rate in genomic DNA (Lee and Chan, 2015). Genome-wide hypomethylation can be specifically reprogrammed by siRNA, indicating a beneficial role of siRNA intervention. Using B1 siRNA topical treatment resulted in DNA damage diminution by specifically methylating B1 elements and enhancing the growth of cells in wound beds, mainly fibroblasts, which eventually accelerated wound healing in diabetes (Table 2). Our findings may prompt the use of B1 (or Alu) siRNA transfection for therapeutic purposes, at least for local treatment, in DNA damage-derived disorders in a preclinical study. Taken together, B1 (or Alu) siRNA intervention may be advantageous to reprogram genome-wide hypomethylation in diabetes and other age-related diseases.

The present study used a calcium-phosphate nanoparticle, which is an inorganic, nonviral vector gene delivery system. We transfected B1 siRNA/Ca-P into target cells by topical administration and demonstrated successful and unharmful transfection *in vivo* (Figure 1). Due to the pre-existence of their composition in cells or tissues, Ca-P nanoparticles encapsulating the genetic material possess biocompatibility, biodegradation, low immunogenic, and low toxicity properties in the transfected cells (Yin et al., 2014; Ramamoorthi and Narvekar, 2015). In addition, we provided an accessible technique for Ca-P nanoparticle coating and preparation with a simple material requirement. Thus, the present study supported that Ca-P nanoparticles are a biologically safe, suitable, and effective gene transfer tool for *in vivo* siRNA transfection treatment.

This study proved that SINE methylation editing by SINE-siRNA can promote DM wound healing. However, the implementation of this technology in humans needs to be cautiously planned. In addition to RdDM, SINE-siRNA may also play an RNA interference (RNAi) role. Alu sequence may be present in some cells in crucial mRNA, and Alu siRNA may cause cell death in some cell types. Another factor that may cause different outcomes between B1 and Alu is genome distribution. However, we did not observe different outcomes in DNA damage reduction between B1-siRNA in rats and Alu-siRNA in human cells. The reason may be due to the DNA protection effect of DNA methylation spreads in very long distances (Mutirangura, 2018).

Although wound treatment complications were not observed, systemic B1 (or Alu) siRNA intervention was precautionary. In addition to DNA methylation, B1 (or Alu) siRNA may be incorporated into the RNA-induced silencing complex (RISC) to regulate mRNA degradation or interfere with protein synthesis. Therefore, B1 (or Alu) siRNA may interfere with the physiological function of some cells. Moreover, B1 and Alu genome distributions are different. Additionally, the present preclinical study consisted of diabetic wound treatment in a rat model; however, the beneficial roles and adverse effects using Alu siRNA, targeting Alu elements in the human genome, by a topical treatment in wound healing with normal or diabetic conditions need to be confirmed in a clinical study.

## Conclusion

In conclusion, the STZ-induced type I diabetic rat model is an appropriate and adequate diabetic animal model for the study of B1 hypomethylation and its consequences. The present study suggested that the STZ-induced rat model shows genome-wide hypomethylation, imitating the diabetic microenvironment milieu by generating and maintaining prolonged hyperglycemia and DNA damage. B1 siRNA intervention in diabetic wounds restores B1 hypomethylation and limits DNA damage, directly accelerating wound repair. B1 hypermethylation by *de novo* DNA methylation results in depletion of DNA damage and ultimately accelerates diabetic wound healing, providing a promising outcome in translational medicine. Hence, these findings underpin the importance of epigenetic reprogramming, particularly DNA methylation editing, as a potential therapeutic target for diabetes and its complications.

## Data Availability

The raw data supporting the conclusion of this article will be made available by the authors, without undue reservation.
