# Supplementary material for: B1 siRNA Increases de novo DNA Methylation of B1 Elements and Promotes Wound Healing in Diabetic Rats
Source: Front Cell Dev Biol. 2022 Jan 19;9:802024. doi: 10.3389/fcell.2021.802024 (PMC8807477; doi:10.3389/fcell.2021.802024)
Supplement: Supplementary file 1 [file DataSheet1.docx]

Supplementary Material


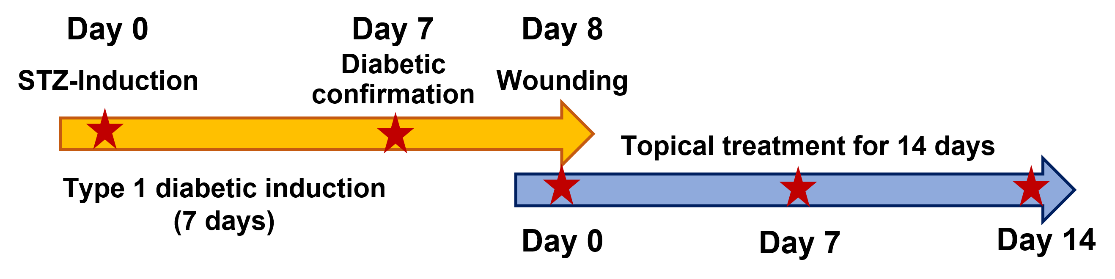


**Supplementary Figure 1.** **Time points of fasting blood glucose measurement.** Five-time points of FBS detection were performed during the study and indicated as the red stars in the timeline. First, after 7-day acclimatization, FBS was measured to be the baseline of the blood glucose level before a diabetic induction. Second, the detection of FBS levels at 7-day of STZ injection was used for differentiation between nondiabetic and diabetic rats, as mentioned in the Materials and Methods. Third, the FBS levels were determined on the day of wounding (day eight after STZ injection, before starting the topical treatment (day 0)). Finally, to confirm the nondiabetic and diabetic conditions in rats, the FBS levels were observed in all groups on day 7 and day 14 (the fourth and the fifth times) after the treatment.

**Supplementary Table 1. The FBS level of nondiabetic and diabetic rats during the study.** The nondiabetic group (FBS<150 mg/dL) was the normal control group. The rats with >250 mg/dL of FBS levels were defined as diabetic rats (n=5 each group). FBS; fasting blood sugar, NSS; normal saline solution, STZ; streptozotocin.

| **Fasting blood glucose (mg/dL)** | **Nondiabetic** | **Diabetic** | |
| --- | --- | --- | --- |
|  |  | **NSS-treated** | **B1 siRNA-treated** |
| 1. Day 0 (baseline) | 113.6 ± 4.03 | 119.2 ± 4.02 | 117.4 ± 4.73 |
| 2. Day 7 after STZ induction | 120.4 ± 3.97 | 382.6 ± 18.88*** | 387.6 ± 14.64*** |
| 3. Day 0 (the day of wounding) | 127.0 ± 5.58 | 448.4 ± 17.79*** | 450.8 ± 17.94*** |
| 4. Day 7 after treatment | 128.6 ± 8.26 | 446.2 ± 25.67*** | 423.8 ± 11.14*** |
| 5. Day 14 after treatment  (the end of study) | 122.6 ± 8.6 | 424.4 ± 18.4*** | 410.8 ± 11.1*** |

Data are presented as means ± S.E.M. *** P < 0.001 significant difference compared with the nondiabetic group.


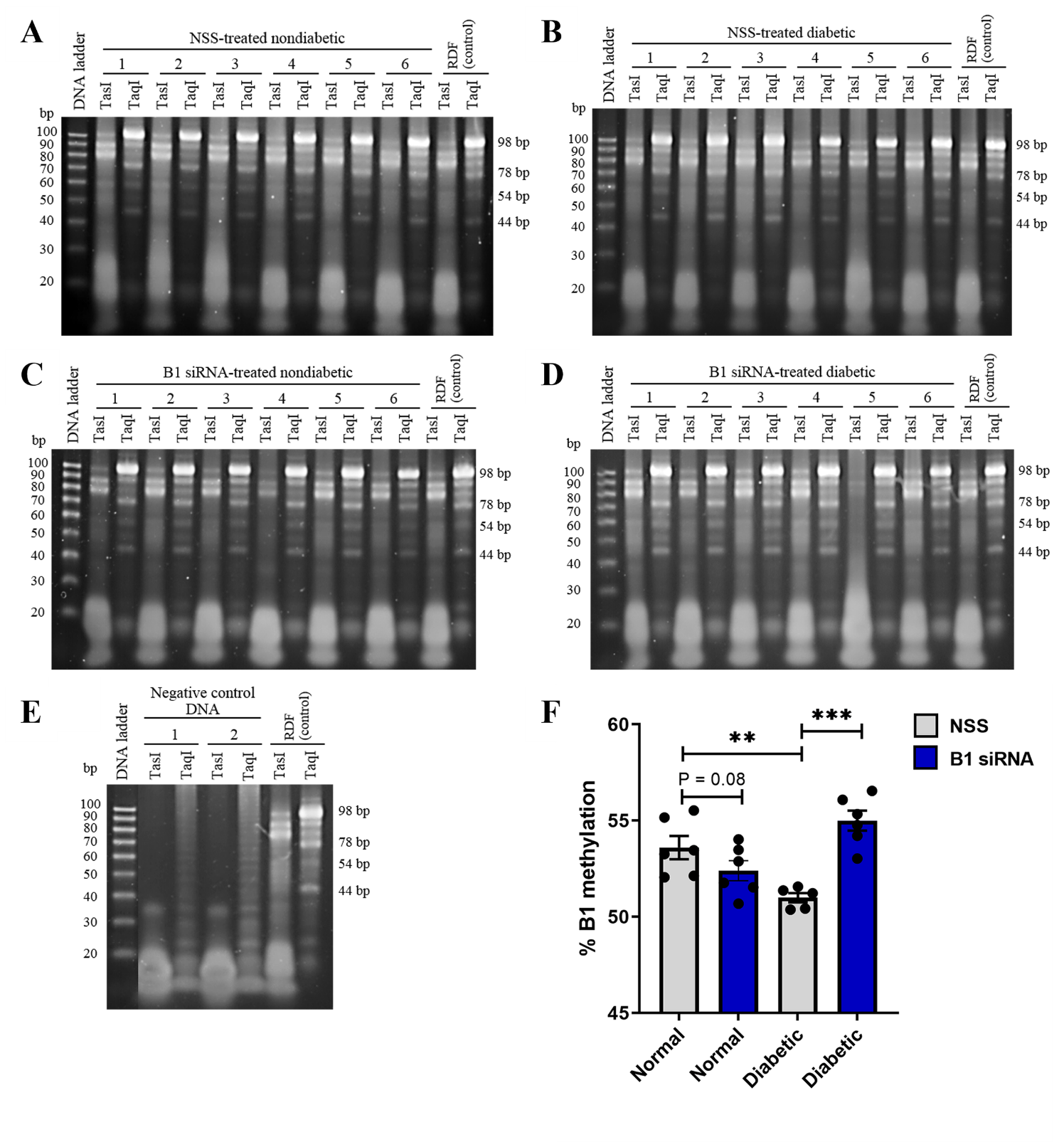


**Supplementary Figure 2. Measurement of B1 methylation by B1-COBRA in rat wound DNA.** Rat wound DNA was extracted and modified by bisulfite before B1-PCR amplification. The B1-PCR products were digested with TagI and subsequently detected by 8% gel electrophoresis. The four target bands (98, 78, 54, and 44 bp) of digested B1-PCR products were determined, and the band intensity of each experimental group, including NSS-treated nondiabetic (**A**), NSS-treated diabetic (**B**), B1 siRNA-treated nondiabetic (**C**), and B1 siRNA-treated diabetic (**D**) rat wound DNA, was analyzed at 14 days after treatment. HEK293 DNA was used as a negative control DNA for B1-PCR analysis (**E**), and rat dermal fibroblast (RDF) DNA was subjected to an inter-assay internal control (**A-E**). Percentage of B1 methylation levels in B1 siRNA-treated (blue bar) wound DNA compared to NSS-treated (gray bar) wound DNA of diabetic and nondiabetic (normal) rats (**F**). Data represent the means ± S.E.M. **P ≤ 0.01 and ***P ≤ 0.001 according to the one-way ANOVA followed by post hoc analysis (n=5-6 per group).


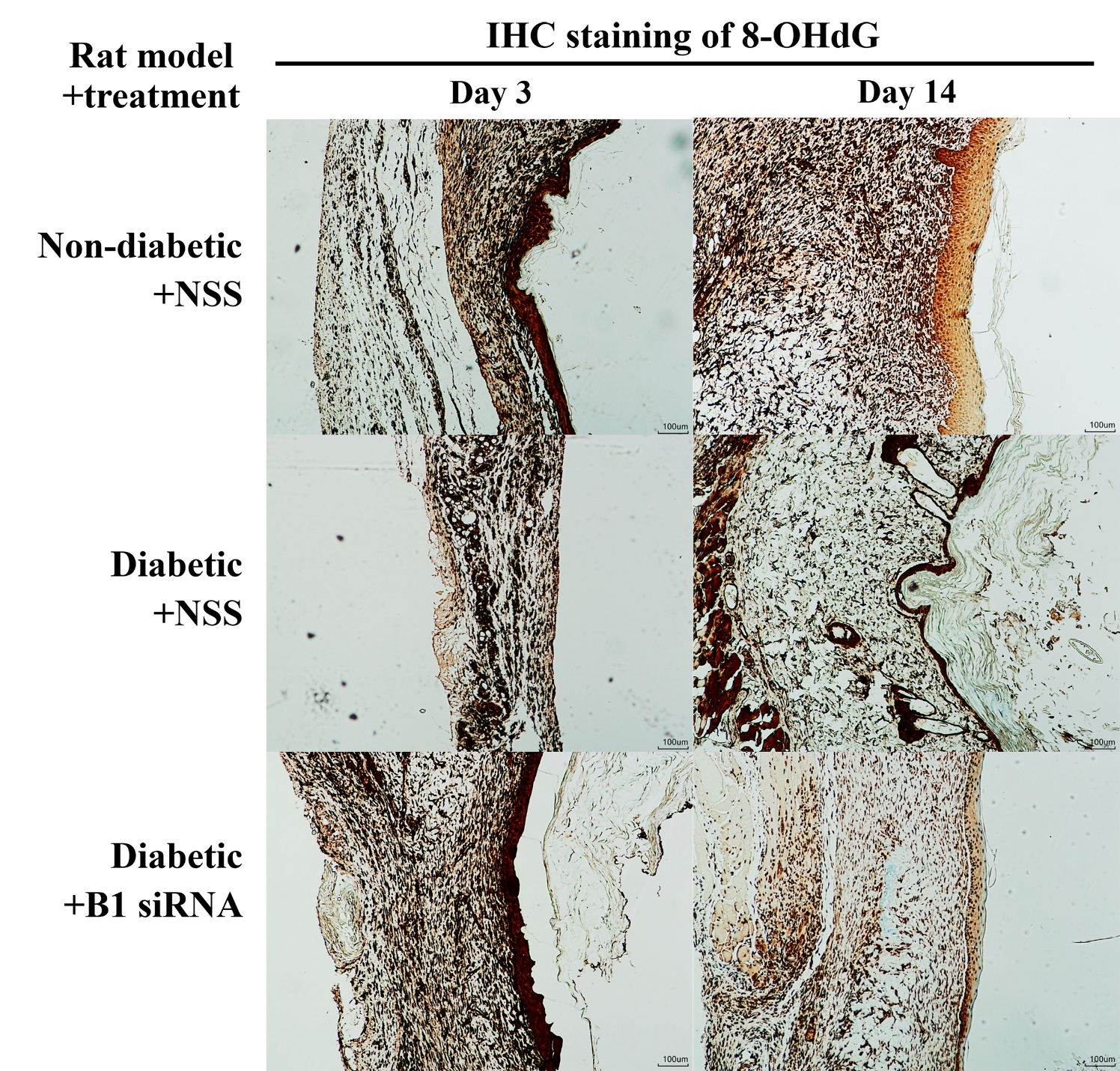


**Supplementary Figure 3. Immunohistochemistry (IHC) staining of the 8-OHdG DNA damage marker.** B1 siRNA transfection showed a decrease in 8-OHdG endogenous DNA damage (brown color) in wound areas of diabetic rats at the end of the study (day 14 after treatment, lower panel) compared to NSS-treated diabetic rats (middle panel).


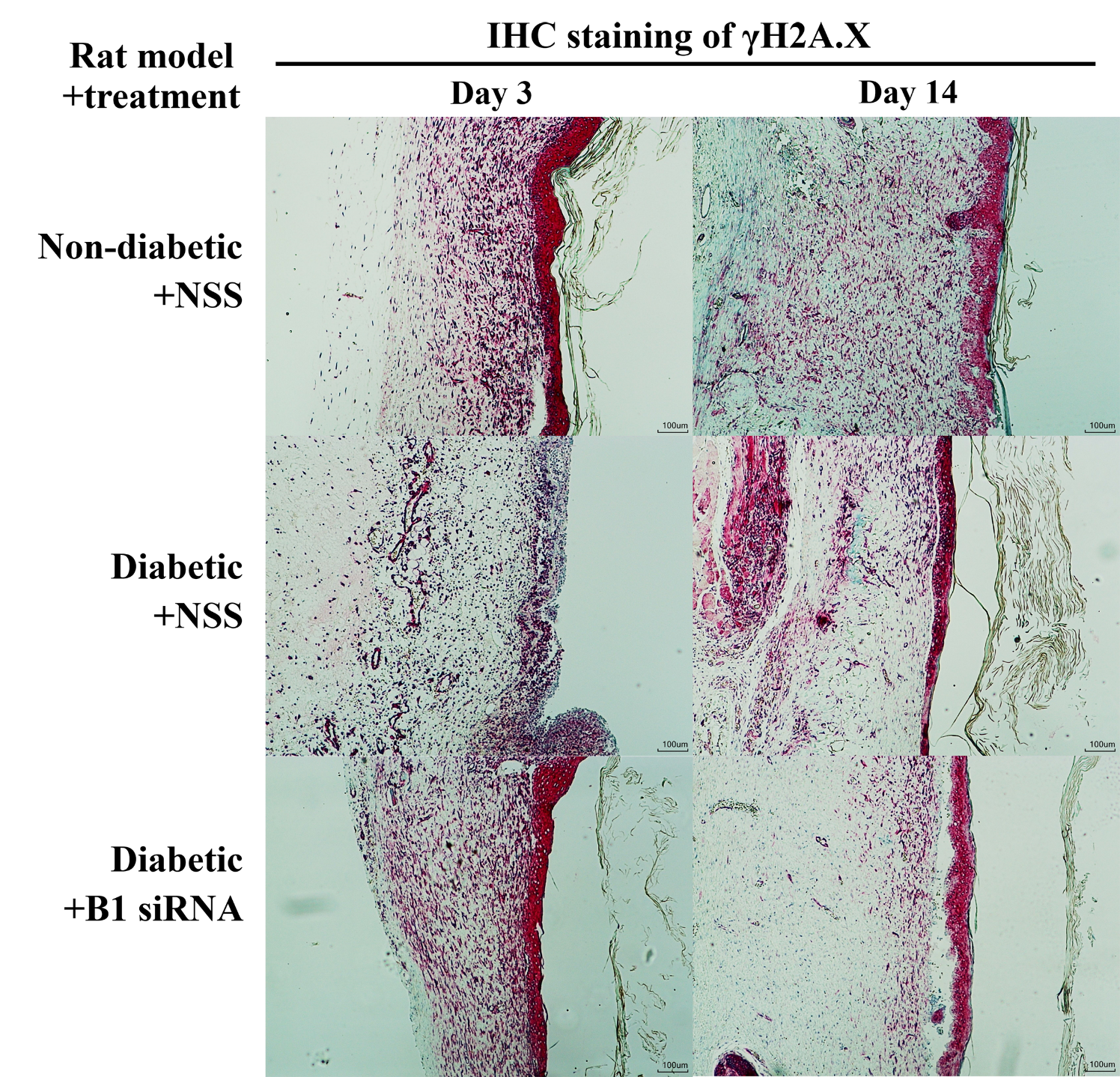


**Supplementary Figure 4. IHC staining of γH2A.X DNA damage marker.** Treatment of B1 siRNA reduced levels of the γH2A.X DNA damage marker (brown color) in wound areas of diabetic rats at the end of the study (day 14, lower panel) compared to NSS-treated diabetic rats (day 14, middle panel).
